# Complete coding sequence of Tembusu virus isolate 2211-5 isolated in Taiwan

**DOI:** 10.1128/mra.00482-24

**Published:** 2024-09-24

**Authors:** Yen-Ping Chen, Chien-Hui Liao, Chih-Wei Huang, Fan Lee

**Affiliations:** 1 Veterinary Research Institute, Ministry of Agriculture, New Taipei City, Taiwan; Katholieke Universiteit Leuven, Leuven, Belgium

**Keywords:** Tembusu virus, coding sequence, Taiwan

## Abstract

We reported the complete coding sequence of a Tembusu virus from sick geese in Taiwan in 2022. The nucleotide sequence of the 2211-5 isolate was most closely related to the strain CTLN isolated from chicken in China and classified into Cluster 3 of Tembusu virus.

## ANNOUNCEMENT

Tembusu virus (TMUV) belongs to the genus *Flavivirus* of the family *Flaviviridae*. Since 2010, TMUV has caused a novel disease characterized by severe drop in egg production and neurological signs in poultry in China. Then, the disease spread to duck farms in Malaysia and Thailand (1–4). Furthermore, a variant TMUV was discovered in mosquitoes and diseased ducks in 2019 and in diseased geese in 2020 in Taiwan (5–7). Here, we report the complete coding sequence of TMUV isolate 2211–5 obtained from a sick goose suffering from diarrhea in Tainan City in southwest Taiwan in 2022.

The 2211–5 isolate was isolated via inoculating the brain homogenate of the diseased goose into the allantonic cavity of three 10-day-old embryonated Muscovy duck eggs (1, 6). Viral RNA was extracted using a MagDEA Dx SV kit (Precision System Science, Chiba, Japan). After constructing of a paired-end sequencing library using Illumina Stranded Total RNA Prep (Illumina, California, USA) according to the manufacturer’s protocol, sequences were generated using a 500-cycle (2 × 250-bp paired-end) MiSeq Reagent Nano kit version 2 (Illumina) with MiSeq sequencer. Base quality lower than Q30 were trimmed using BBDuk (8) implemented in Geneious Prime version 2022 (https://www.geneious.com). *De novo* assembly was conducted using the Geneious assembler. The longest consensus sequence, assembled from 147 reads, was 2,734 bp in length with an average sequence depth of 13.45X. This sequence was identified as TMUV through a BLASTN search. The consensus sequence was compared using default NCBI BLAST settings with highly similar sequences (megablast) and standard databases (9, 10). The most closely related strain (CTLN; MZ355579) was used as the reference sequence for read mapping. The obtained consensus sequence was used to remap the trimmed reads as a final control. Subsequently, the ORF of this strain were aligned (using MAFFT version 7) together with other selected TMUV strains (11, 12). A maximum-likelihood phylogeny was reconstructed using the Tamura–Nei substitution model (13) in Molecular Evolutionary Genetics Analysis version X (14) with 1,000 replicates of a bootstrap test.

**Fig 1:**
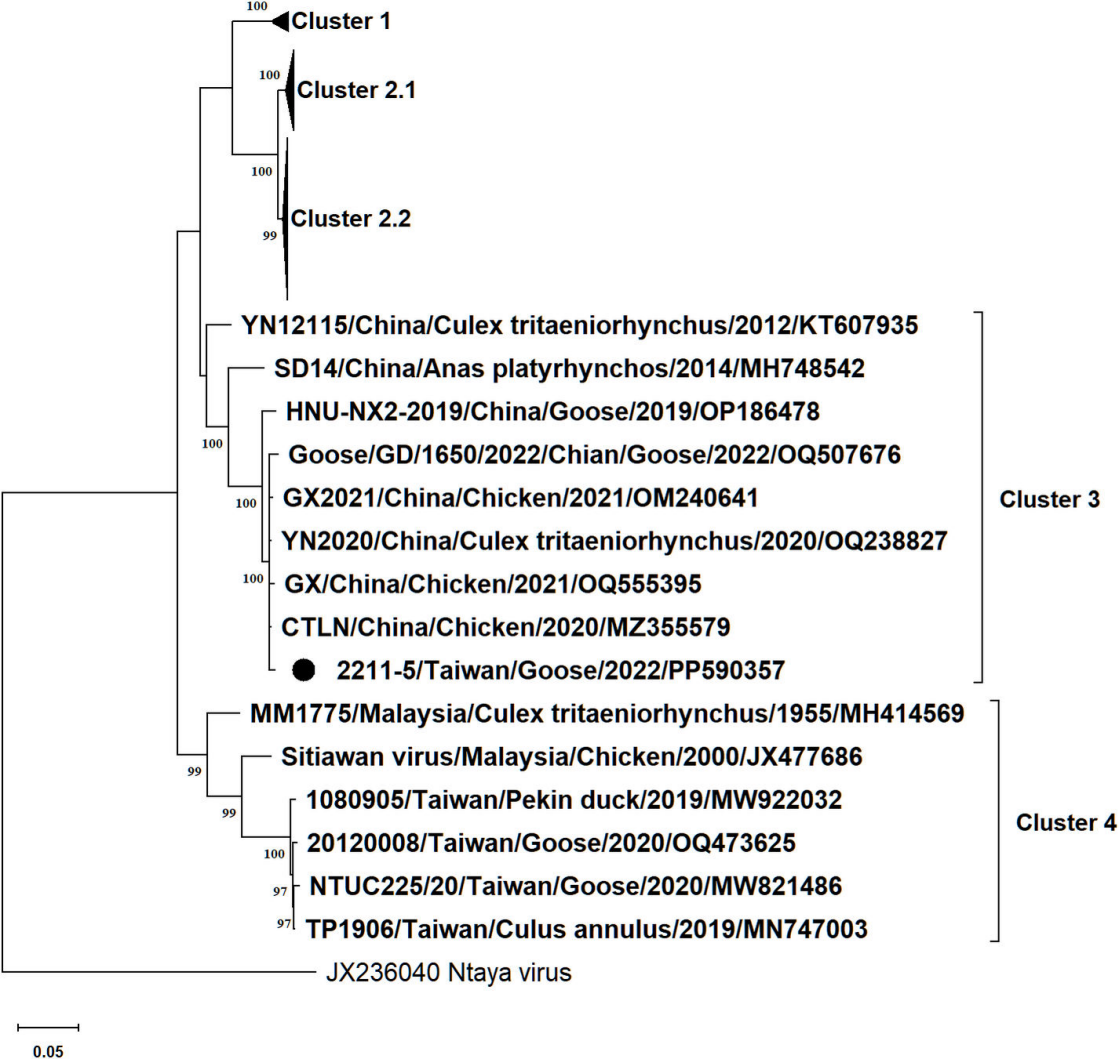
Maximum-likelihood phylogeny of Tembusu virus based on the complete polyprotein nucleotide sequences. Only bootstrap values (1,000 replicates) over 70% are indicated at each branch point as a percentage. For each virus strain, strain name, country, host, year of isolation or detection, and GenBank accession number are shown. The sequence of Tembusu virus 2211-5 isolate is indicated with a black solid circle.

In total, 5,927 of 1,042,552 reads were assembled to the genome of the TMUV 2211–5 isolate. The final consensus sequence was 10,975 bp with 49.1% GC content and contained a single ORF encoding a polyprotein comprising 3,425 amino acids, a 86-bp 5’ untranslated region (UTR), and a 611-bp 3’ UTR. The average sequencing depth was 269.79X. Comparing to the TMUV CTLN strain, our sequence missed a 9-bp sequence in the 5’ UTR and 11-bp sequence in the 3’ UTR. The ORF sequence alignments revealed that the 2211–5 isolate shared 87.32% to 99.43% nucleotide identities with the TMUV strains isolated in China, Thailand, Malaysia, and Taiwan, while it displayed the greatest similarity (99.43%) to the TMUV CTLN strain isolated in China. The phylogeny showed that the 2211–5 isolate was classified into Cluster 3 that consisted of Chinese TMUVs, and, interestingly, not classified into Cluster 4 to which the previously reported Taiwanese TMUVs belonged (Fig. 1).

The reported data contributes to the exploration of the molecular epidemiology and evolutionary of the TMUV.

## Data Availability

Raw reads were deposited in SRA under accession number PRJNA1100215. The assembled genomic sequence of the TMUV 2211–05 isolate has been deposited in GenBank under accession number PP590357.
